# Supplementary figures and images for: The Notch1 intracellular domain orchestrates mechanotransduction of fluid shear stress
Source: Life Sci Alliance. 2026 Feb 5;9(4):e202503599. doi: 10.26508/lsa.202503599 (PMC12877406; doi:10.26508/lsa.202503599)

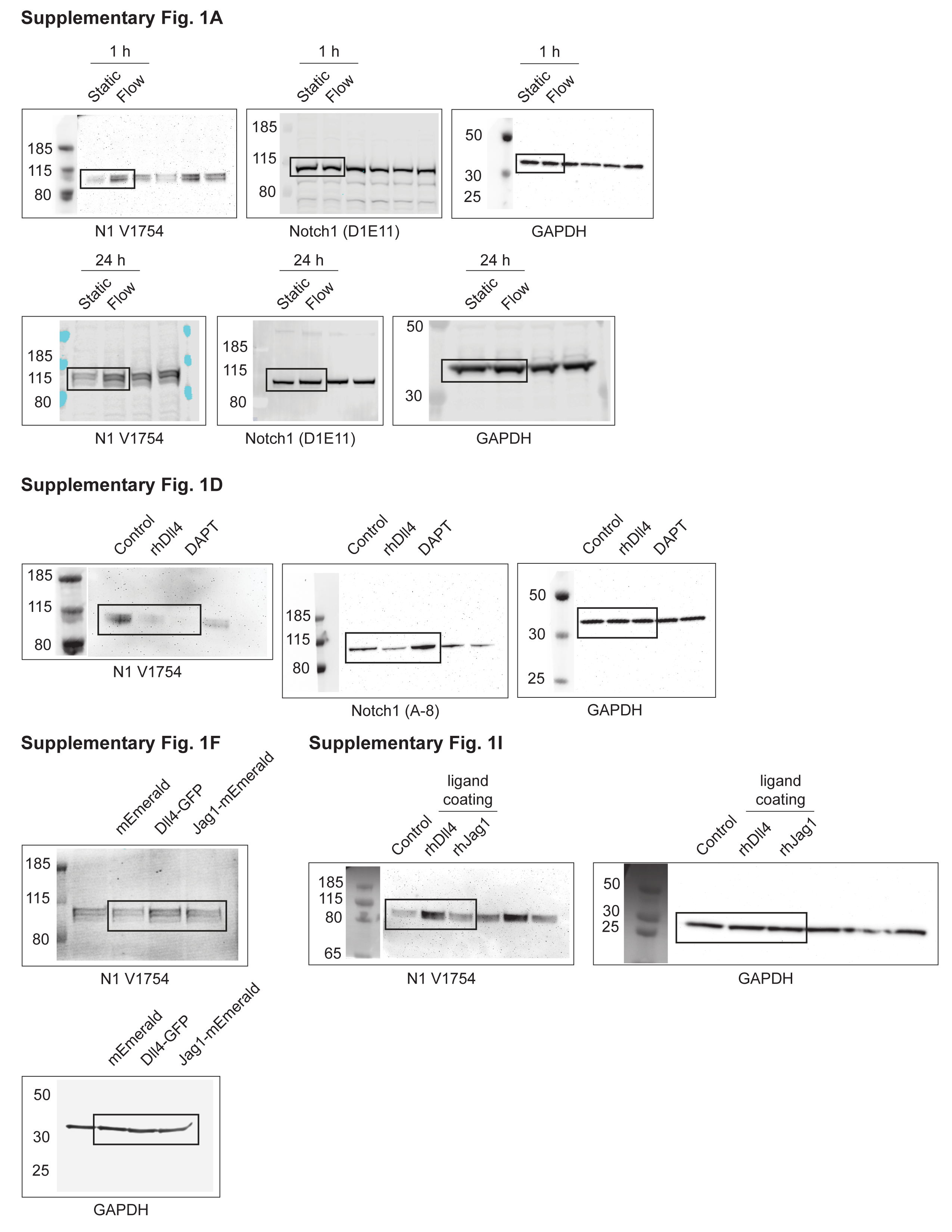

Supplement: Supplementary file 1 [file LSA-2025-03599_SdataFS1.tif]

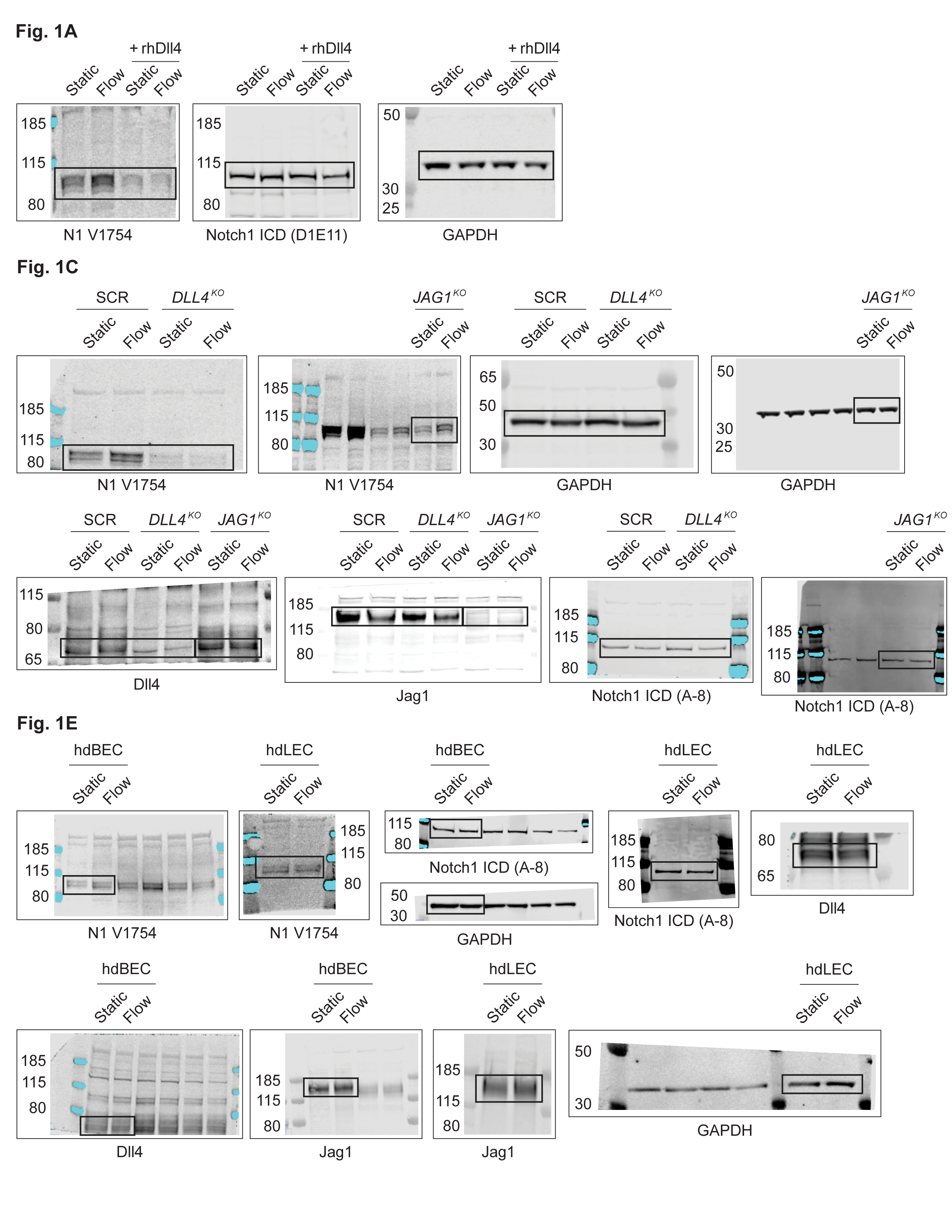

Supplement: Supplementary file 2 [file LSA-2025-03599_SdataF1.tif]

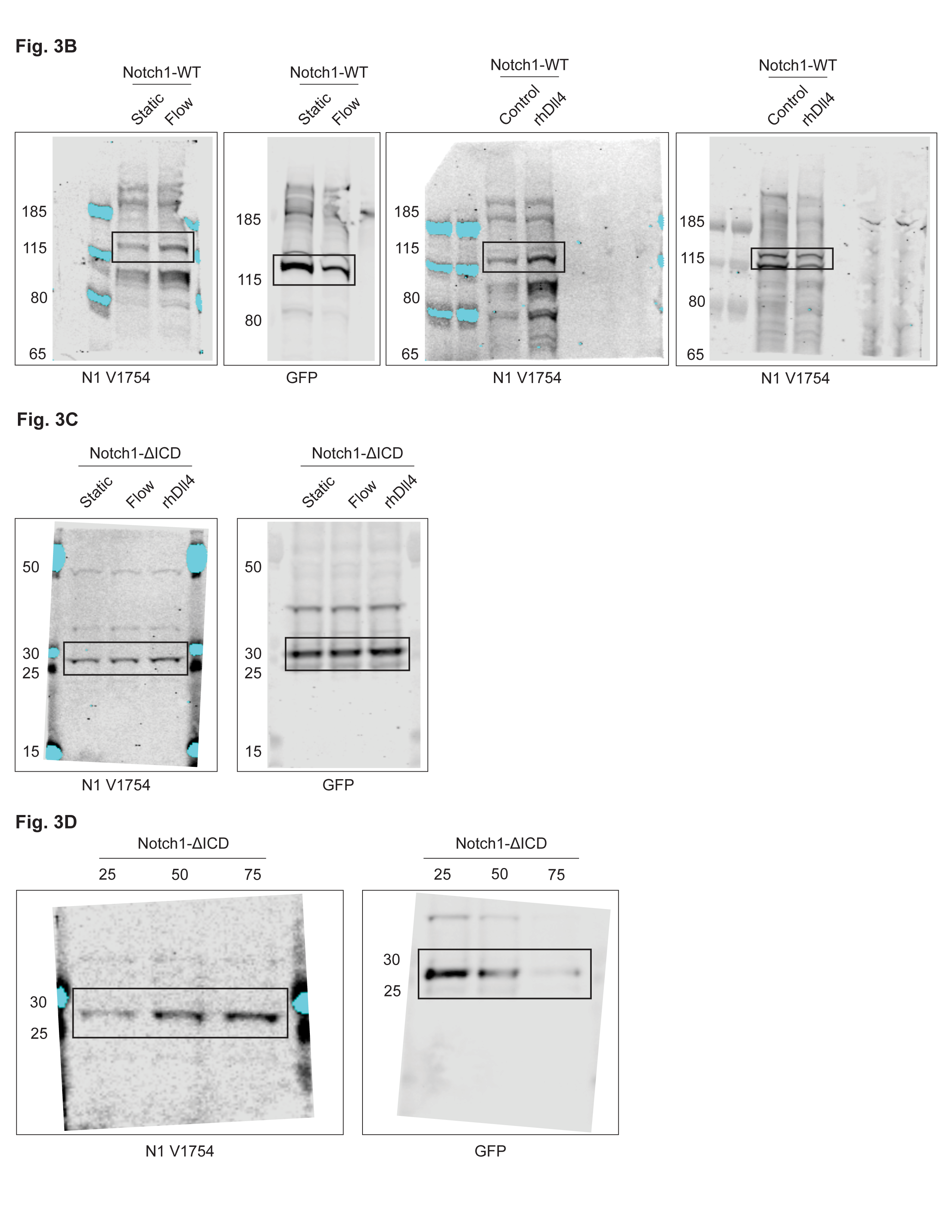

Supplement: Supplementary file 5 [file LSA-2025-03599_SdataF3.tif]

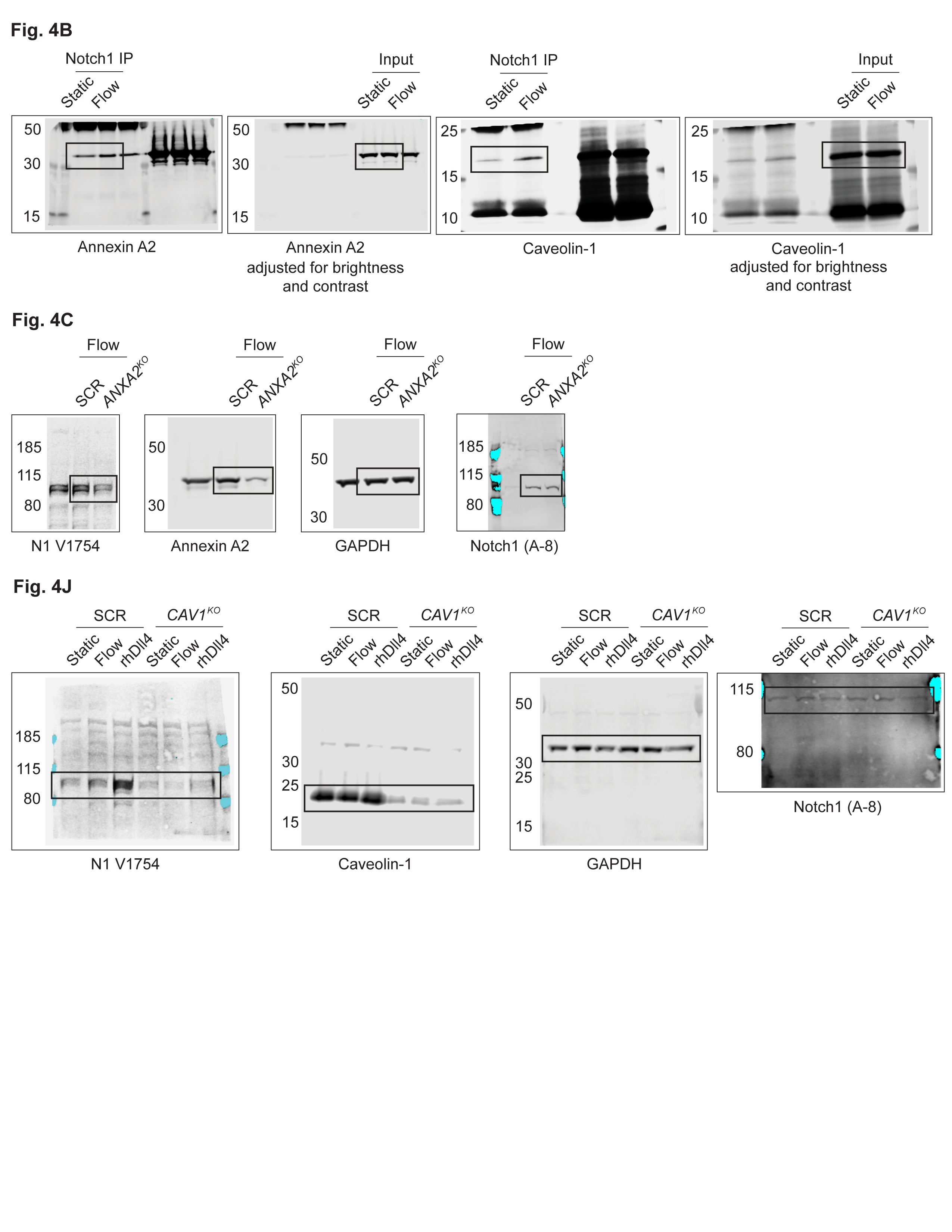

Supplement: Supplementary file 6 [file LSA-2025-03599_SdataF4.tif]
